# Establishment of 3D Neuro-Organoids Derived from Pig Embryonic Stem-Like Cells

**DOI:** 10.3390/ijms22052600

**Published:** 2021-03-05

**Authors:** Seon-Ung Hwang, Kiyoung Eun, Mirae Kim, Junchul David Yoon, Lian Cai, Hyerin Choi, Dongjin Oh, Gabsang Lee, Hyunggee Kim, Eunhye Kim, Sang-Hwan Hyun

**Affiliations:** 1 Laboratory of Veterinary Embryology and Biotechnology (VETEMBIO), Veterinary Medical Center and College of Veterinary Medicine, Chungbuk National University, Cheongju 28644, Korea; ghkdsun@hanmail.net (S.-U.H.); kmr9309@naver.com (M.K.); jdyoon86@gmail.com (J.D.Y.); cailian002@daum.net (L.C.); hyrin3642@naver.com (H.C.); rosecafes123@naver.com (D.O.); 2 Institute of Stem Cell & Regenerative Medicine (ISCRM), Chungbuk National University, Cheongju 28644, Korea; 3 Institute of Animal Molecular Biotechnology, Korea University, Seoul 02841, Korea; eky102@naver.com (K.E.); hg-kim@korea.ac.kr (H.K.); 4 Department of Biotechnology, School of Life Sciences and Biotechnology, Korea University, Seoul 02841, Korea; 5 Department of Neurology, Institute for Cell Engineering, Johns Hopkins University School of Medicine, Baltimore, MD 21205, USA; glee48@jhmi.edu

**Keywords:** porcine, neuro-organoid, embryonic stem cells, somatic cell nuclear transfer, neural differentiation

## Abstract

Although the human brain would be an ideal model for studying human neuropathology, it is difficult to perform in vitro culture of human brain cells from genetically engineered healthy or diseased brain tissue. Therefore, a suitable model for studying the molecular mechanisms responsible for neurological diseases that can appropriately mimic the human brain is needed. Somatic cell nuclear transfer (SCNT) was performed using an established porcine Yucatan EGFP cell line and whole seeding was performed using SCNT blastocysts. Two Yucatan EGFP porcine embryonic stem-like cell (pESLC) lines were established. These pESLC lines were then used to establish an in vitro neuro-organoids. Aggregates were cultured in vitro until 61 or 102 days after neural induction, neural patterning, and neural expansion. The neuro-organoids were sampled at each step and the expression of the dopaminergic neuronal marker (TH) and mature neuronal marker (MAP2) was confirmed by reverse transcription-PCR. Expression of the neural stem cell marker (PAX6), neural precursor markers (S100 and SOX2), and early neural markers (MAP2 and Nestin) were confirmed by immunofluorescence staining. In conclusion, we successfully established neuro-organoids derived from pESLCs in vitro. This protocol can be used as a tool to develop in vitro models for drug development, patient-specific chemotherapy, and human central nervous system disease studies.

## 1. Introduction

Studies of human neuropathologies are hindered by limitations of in vitro culture of the human brain for genetically engineering healthy or diseased brain tissue. To overcome this challenge, many researchers have been developing animal models of human diseases. Recent technological progress has enabled the genetic manipulation of large animals. Pigs, which are anatomically and physiologically similar to humans, have attracted interest in this regard because of their advantages compared to rodents as models in human disease studies [1,2]. Additionally, pig models for brain imaging and neurosurgery studies are increasingly required because of the anatomical similarities between humans and pig [3]. In particular, as a neurological disease model, pigs share higher central nervous system (CNS) similarity with humans than with any other mammal. Both the human and porcine cerebral cortex possess a gyrencephalic brain, which shows similarities with respect to both the character and distribution to the brain gyri (the cortical grey matter), gray matter, and white matter. The pig hippocampus, basal ganglia, and brainstem are also topographically similar to those in humans. Thus, pigs are an ideal animal for preparing a human CNS disease model.

Pluripotent stem cells (PSCs) can be divided into naive and primed pluripotent states depending on their characteristics. The naïve pluripotent state is similar to that of the inner cell mass and is known to be capable of dome-shape colonization, single-cell cloning, leukemia inhibitory factor-dependence, and ability to produce whole animals through tetraploid complementation. The primed pluripotent state is similar to that of epiblast-derived stem cells (EpiSCs) after implantation, but shows molecular and functional differences from embryonic stem cells (ESCs). The characteristics include flat colonies, difficulty in single-cell cloning, activin/fibroblast growth factor-dependence, and inability to produce whole animals through tetraploid complementation. Representative examples include mouse EpiSCs, human ESCs, and porcine ESCs [4].

Recent studies have examined brain organoids derived from PSCs such as ESCs and induced pluripotent stem cells (iPSCs). In 2008, Eiraku et al. reported cultured mouse ESCs using the serum-free floating culture of embryoid body (EB)-like aggregates with a quick reaggregation (SFEBq) method to form large telencephalic rosettes. This method can effectively recreate the process of in situ generation by taking advantage of the self-organization of cell groups, enabling the formation of cortical neuronal epithelial cells in a spatially and temporally controlled pattern rather than as a specific neural network differentiation. Because cortical tissues induced by the SFEBq method form self-organized long-range neural networks, they are more useful for studying the pathogenesis of brain diseases, drug discovery, and regenerative medicine compared to isolated neurons [5]. In 2011, researchers formed fully stratified retinal organoids mimicking in vivo development by culturing mouse ESCs using an approach based on the SFEBq method. In this case, floating culture was used rather than plating on a coated dish, and a modified medium formulation was used to promote retinal development. As a result, the organoids formed were very similar to developing retinas. This study confirmed that nerve tissue maintained in three-dimensional (3D) floating culture can organize and develop a systematically accurate structure [6]. In 2013, Lancaster et al. reported whole-brain organoid formation similar to human brain tissue from human PSCs. They used a modified SFEBq method to create a cerebral organoid model, known as a “mini brain,” and did not use region-specific patterning factors. Matrigel was used as an extracellular scaffolding matrix to grow and expand the neural epithelium, resulting in self-organization and development into various brain regions [7]. More recently, Qian et al. [8] developed human iPSCs for 7 days to form patterned EBs with region-specific patterning factors, which were coated on Matrigel for 7 days and then mass-cultured in a small rotating bioreactor. This approach improved organoid culture reproducibility and successfully produced other brain area-specific organs, including the whole brain, midbrain, and hypothalamic organisms [8]. Recently, there has been a report that a pig embryonic germ cells (pEGCs) line was used to form neuronal precursor cells. However, this was analyzed by seeding on the plate after the neural induction (NI) step [9]. This result is an early stage of neuronal differentiation and cannot be called 3D neuro-organoids.

To induce the formation of such organoids, PSCs such as ESCs and iPSCs are required. These cells are pluripotent and can differentiate into various cell types. ESCs originate from the inner cell mass of the embryo before transplantation, whereas iPSCs are formed through the reprogramming of somatic cells. Although there are various advantages and disadvantages of each cell line, iPSCs have been reported to increase the risk of tumor formation compared to ESCs [10]. Additionally, mitochondrial DNA (mtDNA) damage was increased in iPSCs established using cell lines from elderly patients compared to those derived from younger patients. Structural damage to these mtDNA genes can reduce the energy required for differentiated cells, thereby limiting their therapeutic potential and affecting their application in disease modeling or drug screening. ESCs from elderly patients prepared by somatic cell nuclear transfer (SCNT) can use mitochondria derived from a young donor embryo to overcome the limitation of induced mtDNA damage [11,12].

The midbrain is part of the brainstem. If a brain tumor in this area is surgically removed, the patient can be in a vegetative state. Thus, for brain tumors in the brainstem, chemotherapy may be considered after radiotherapy; however, the prognosis of these patients remains very poor. Additionally, widespread neurological conditions such as Parkinson disease, Huntington disease, and Alzheimer disease are characterized by abnormal hyperactivity of the neurons in the midbrain, which is part of the CNS. Therefore, in vitro midbrain models are urgently needed for drug development and the design of patient-specific chemotherapy as well as fundamental human CNS diseases studies.

Transgenic (TG) model is very useful for tracking the role of specific genes in disease. However, there are ethical barriers to transforming human cells. Pigs are a very good model for human neuro-disease research. However, the production of in vivo pig models is time consuming and expensive. We performed SCNT using pig TG cell lines as well as established TG porcine ES-like cell (pESLC) lines using the SCNT embryos. A 3D neruro-organoid protocol was developed using SCNT pESLC lines. pESLC lines show similar characteristics to human ESCs. In this study, we tried to create a basis for studying human CNS diseases in vitro model using TG pig cells.

## 2. Results

### 2.1. Development of TG Embryos Produced by SCNT

**Figure 1:**
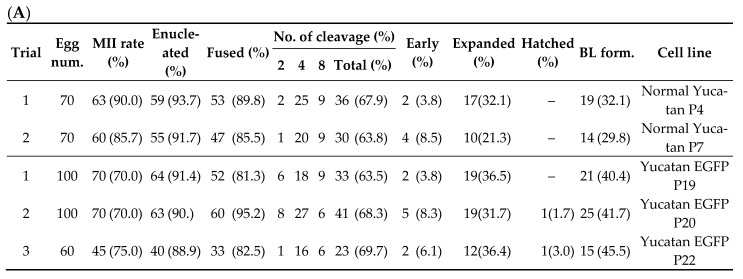

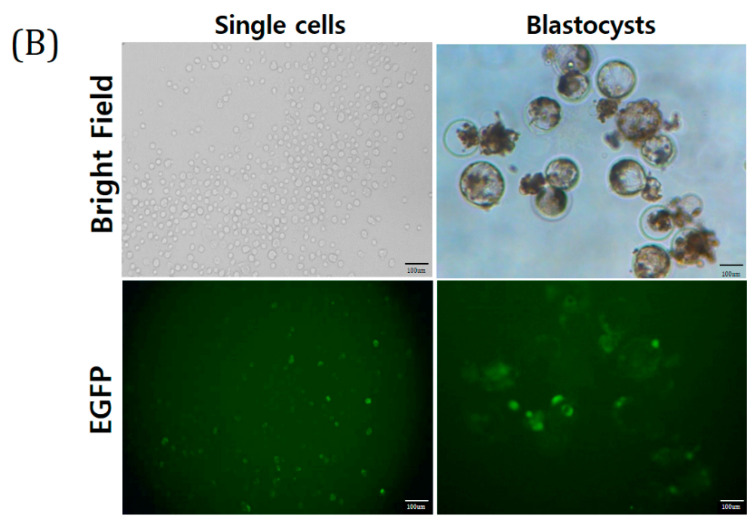
Evaluation of (**A**) cleavage and the blastocyst formation rate of embryos produced through somatic cell nuclear transfer (SCNT). The cleavage rate was measured on day 2, and blastocyst formation rate was evaluated on day 7 of culture. (**B**) Expression of enhanced green fluorescent protein (EGFP) in TG donor cell and SCNT BLs. Scale bars 100 µm.

SCNT was performed using Normal Yucatan and Yucatan EGFP cell line as the donor cell line. Yucatan, used as a donor cell, is a type of mini pig. On the 2nd day of IVC, the cleavage (CL) rate of TG embryos was compared with normal data, and there was no negative effect. On the 7th day of IVC, the TG blastocyst (BL) formation rate was compared with normal data, and there was no negative effect (Figure 1A). Enhanced green fluorescent protein (EGFP) expression was confirmed by single cells, and BLs by microscopy (Figure 1B).

### 2.2. Establishment and Morphological Analysis of pESLCs Derived from SCNT BLs

**Figure 2:**
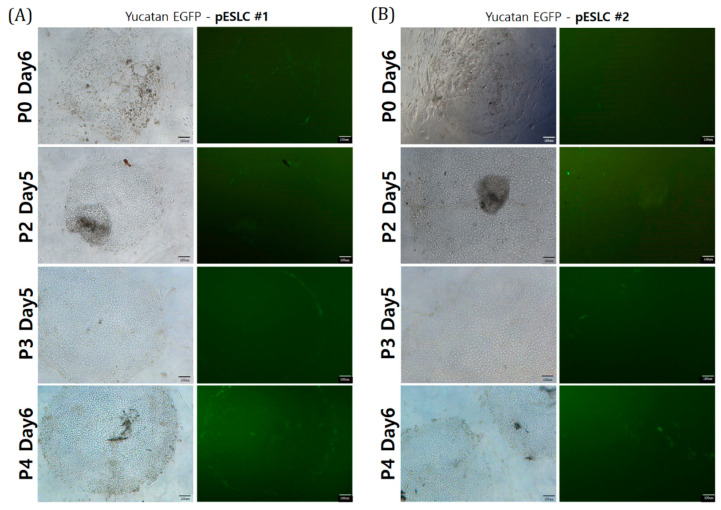
Morphology and EGFP expression of two pESLC lines (**A**,**B**). The pESLC lines derived from SCNT embryos from a Yucatan donor cell with EGFP. Scale bars 100 µm.

The BLs with the EGFP gene produced by SCNT were seeded onto MEF feeder cells. On the 7th day after seeding, two primary growths were observed from SCNT BL derived from Yucatan EGFP P19. Colonies from the early passage had an irregular colony morphology and uneven colony margin and showed a low cell density, representing a mixed pattern of differentiated cells and pESLCs. However, with increasing passaging of the cell line, the colonies had a regular morphology, even margin, and higher cell density, demonstrating typical characteristics of common pESLCs. Morphologically, two pESLC lines with a primed pluripotent state morphology were established. Expression of EGFP was mainly observed at the margin (Figure 2).

### 2.3. Characterization of Yucatan EGFP pESLC Lines

**Figure 3:**
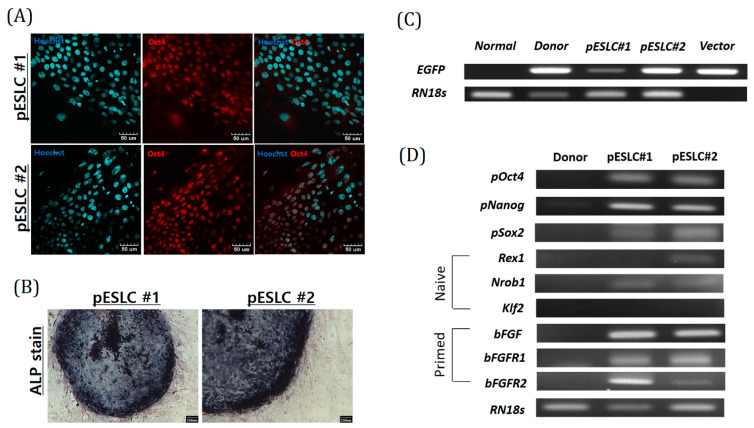
Characterization of Yucatan EGFP pESLC lines. (**A**) The expression of pluripotent marker (Oct4) in Yucatan EGFP pESLC lines as assessed by immunofluorescence analysis; scale bars = 30 mm. (**B**) Alkaline phosphatase (ALP) stain; scale bars = 100 mm. (**C**) Identification of transgenes in genomic DNA from the Yucatan EGFP pESLC lines by polymerase chain reaction (PCR). *RN18s* was used as a control. (**D**) The expression of pluripotent marker genes (undifferentiated pluripotent cell marker: *pOct4*, *pNanog*, and *pSox2*; naive markers: *Rex1*, *Nrob1*, and *Klf2*; and primed markers: *bFGF*, *bFGFR1*, and *bFGFR2*) in Yucatan EGFP pESLCs.

The characteristics of the two established Yucatan EGFP pESLC lines were analyzed. First, expression of the pluripotent marker OCT4 in the pESLC line was confirmed by immunofluorescence staining (Figure 3A). Alkaline phosphatase (ALP) staining further showed that both pESLC lines had ALP activity in the colonies except for those in MEF feeder cells (Figure 3B). Reverse transcription (RT)-PCR evaluation for the expression of naïve (*Rex1*, *Nrob1*, and *Klf2*) and primed (*bFGF*, *bFGFR1*, and *bFGFR2*) pluripotent state markers confirmed the expression of the primed pluripotent state marker in both pESLC lines. In particular, the expression of the naïve pluripotent state marker *Rex1* was partially confirmed in pESLC line #2. In addition, the undifferentiated pluripotent cell markers *pOct4*, *pNanog*, and *pSox2* were identified in both pESLC lines (Figure 3D). Additionally, the presence of the inserted EGFP gene was confirmed by PCR using gDNA. The EGFP gene was present in the two pESLC lines (Figure 3C). These findings confirmed the successful insertion of EGFP gene and expression of pluripotency markers.

### 2.4. Differentiation Potential of Yucatan EGFP pESLC Lines

**Figure 4:**
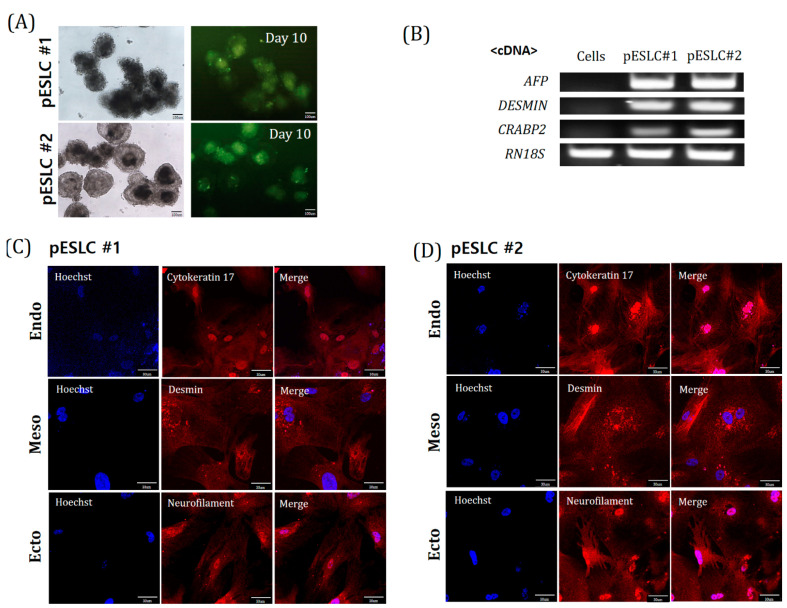
Differentiation potential analysis in Yucatan EGFP pESLC lines. (**A**) Morphology and EGFP expression of embryoid bodies (EBs) derived from pESLC lines. Scale bars = 100 mm. (**B**) The expression of marker genes *Alpha-fetoprotein* (*AFP*; endoderm), *Desmin* (mesoderm), and *Cellular retinoic acid binding protein 2* (*CRABP2*; ectoderm) involved in differentiation in the EBs. RN18s was used as a control. (**C**,**D**) The expression of the differentiation marker Cytokeratin 17 (endoderm), Desmin (mesoderm), and Neurofilament (ectoderm) from EBs by IF analysis. Scale bars = 30 mm.

EB formation was induced to confirm the ability of the established cell lines to differentiate into the three germ layers of the established pESLC lines. On the 10th day, spherical EBs expressing EGFP were formed (Figure 4A). The expression of the three-layer markers *AFP* (endoderm), *DESMIN* (mesoderm), and *CRABP2* (ectoderm) was confirmed by RT-PCR in all EBs derived from Yucatan EGFP pESLC lines (Figure 4B). Expression of Cytokeratin 17 (endoderm), Desmin (mesoderm), and Neurofilament (ectoderm) in both pESLC line-derived embryos was confirmed by immunofluorescence (Figure 4C,D). These results confirmed the differentiation potential of the pESLC lines into the three germ layers.

### 2.5. Generation of Neuro-Organoids from Yucatan EGFP pESLC Lines

**Figure 5:**
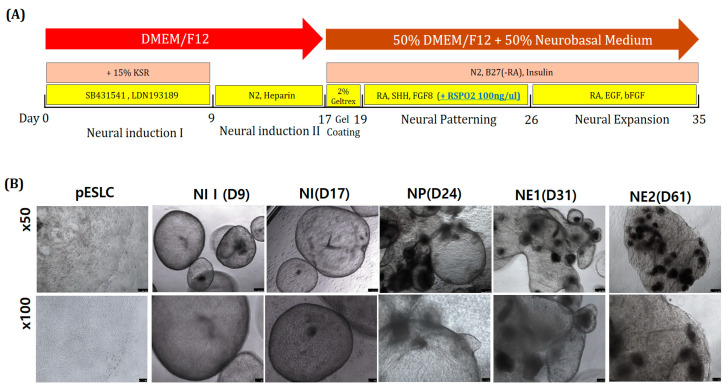
Progression of 3D neuro-organoid development from Yucatan EGFP pESLCs. (**A**) Schematic diagram of the 3D neuro-organoid method and timing. (**B**) Image of neuro-organoid according to differentiation stage. ×50: scale bar = 250 μm; ×100: scale bar = 100 μm.

**Figure 6:**
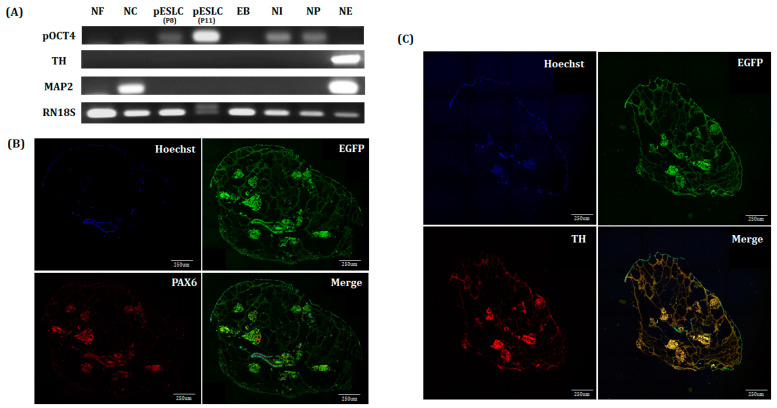

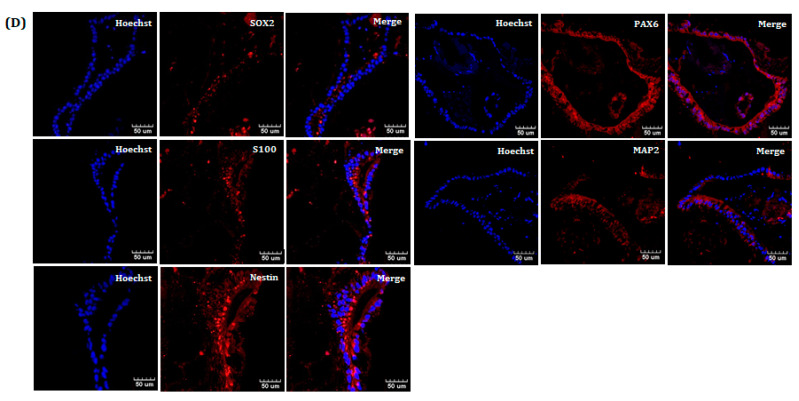
Characterization of D61 neuro-organoid from Yucatan EGFP pESLC lines. (**A**) Analysis of gene expression by differentiation step by PCR. RN18s was used as a control. NF: normal fibroblast, NC: neural cell, EB: embryonic body, NI: neural induction, NP: neural patterning, NE: neural expansion. (**B**–**D**) Immunofluorescence staining of neuro-organoids. Scale bar = 50 or 250 μm.

Porcine neuro-organoid cultures were carried out by modifying the human brain organoid culture protocol and the neural differentiation and culture protocol of piPSCs (Figure 5A). Neuro-organoids were formed in the NI, NP, and NE stages and cultured in vitro for up to 61 days. The morphological changes of the neuro-organoids were observed at each step. First, when inducing neural differentiation (NI stage), the neuro-organoids were expanded to about 2 mm in diameter. After the gel-coating step, the neuro-organoids bonded to each other at the NP stage. In the NE stage, each of the bonded neuro-organoids formed as a large neuro-organoid (Figure 5B). Expression of the dopaminergic neuronal marker TH and mature neuronal marker MAP2 was confirmed on day 61 by RT-PCR. Expression of MAP2 was also confirmed in neural cell (Figure 6A). On day 61, the neuro-organoid was cryosectioned and immunofluorescence stained. As a result, the whole neuro-organoid consisted of several small individual neuro-organoids, which were connected to cells expressing EGFP. In addition, PAX6, a neural stem cell marker, and TH, dopaminergic neuronal marker, were expressed in individual neuro-organoids (Figure 6B,C). In addition, expression of MAP2, the neural stem cell marker PAX6, neural progenitor markers S100 and SOX2, and the early neuronal marker Nestin was confirmed by enlarging individual neuro-organoids (Figure 6D).

### 2.6. Long-Term Culture of Neuro-Organoids from Yucatan EGFP pESLC Lines

**Figure 7:**
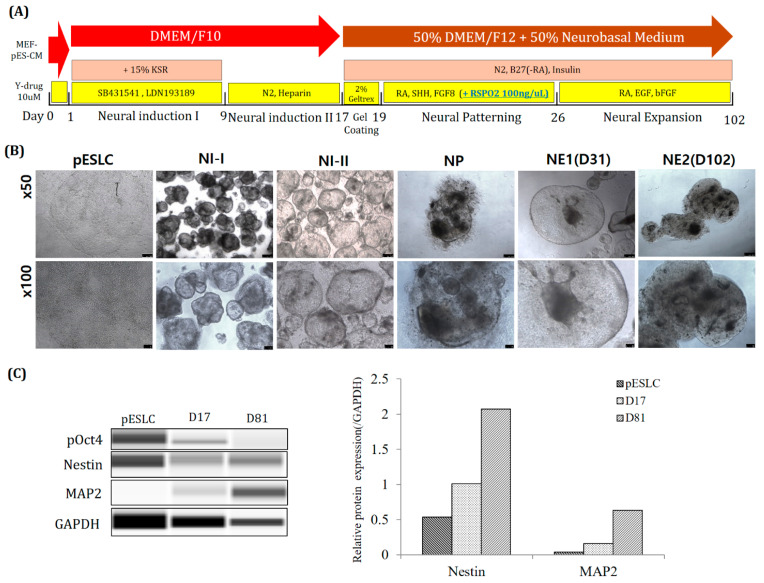
(Long-term) Progression of 3D Neuro-organoid development from Yucatan EGFP pESLCs. (**A**) Schematic diagram of the 3D neuro-organoid method and timing. (**B**) Image of neuro-organoid according to differentiation stage. Scale bar: ×50 = 250 um, ×100 = 100 um. (**C**) Result of Western blot.

For long-term culture, Y-27632 and MEF-CM were used for the first day. In addition, for NI medium, low-glucose DMEM and F10 were used (Figure 7A). As a result, neuro-organoid culture was possible up to 102 days (Figure 7B). In addition, as a result of Western blot analysis, the expression of Nestin and MAP2 proteins tended to increase (Figure 7C).

## 3. Discussion

Recently, an increasing number of studies on brain organoids derived from PSCs have been performed, such as ESCs and iPSCs. For establishment of an in vitro model, in this study, we established two pESLC lines by whole seeding of SCNT BLs. Previously, pESLC lines were reported to have a primed pluripotent state [13]. pESLC lines were established with this primed pluripotent state and their pluripotent and three germ-layer differentiation potential was confirmed. The established pESLC line was then used for suspension culture in NI medium to form neuro-organoids. Most researchers have used dual-SMAD inhibition for NI [8,14,15]. In experiments with human ESCs, dual-SMAD inhibition promoted nerve development in the natural ectoderm through inhibition of bone morphogenic protein by LDN193189, and endogenous actin and nodal signals were inhibited by SB431542, thereby inducing rapid and complete neurotransmission [16]. However, induction of differentiation using specific factors does not result in the formation of a whole-brain organoid. Recently, Lancaster et al. [7] reported the formation of a whole-brain organoid without using specific pattern factors. In the present study, pattern factors were used in the first stage of NI to induce free differentiation, but no specific pattern factors were used in the second stage of NI. In the gel-coating step, 2% Geltrex was mixed with DN medium. Basement membrane matrix products such as Matrigel and Geltrex function as substrates or physical supports for cultured cells to help produce more *in vivo*-like extracellular matrices. Most previous studies did not use this basement membrane matrix. However, this process has recently been used to produce the most advanced form of the whole brain, forebrain, midbrain, or hypothalamus organoids [7,8]. The brain is composed of various nerve cells, and differentiation depends on the environment. Neural stem cells may remain in the complex, resulting in continuous differentiation. Therefore, Geltrex was used to form one neuro-organoid from several neuro-organoids (Figure 5B). After incubation for up to 61 days, the neuro-organoids were confirmed to be organically linked with each other according to Immunofluorescence staining (Figure 6B,C).

To induce midbrain differentiation, SHH, FGF8, and RSPO2 were added to the NP-stage culture medium. Previous studies showed that dual SMAD, SHH, FGF8, and WNT signals are important for neural tube differentiation during neural tube development [17]. A recent study further indicated that RSPO2 treatment promotes midbrain dopaminergic neurogenesis and differentiation in the neural differentiation process of mouse ESCs, which known as the naïve state, and in human ESCs, which is known as the primed pluripotent state [18]. The RSPO family has been reported to be associated with WNT signals from other tissues such as intestinal stem cells and Paneth cells [19]. Additionally, RSPO2 promotes proliferation and migration through the WNT/β-catenin pathway in human hepatocellular carcinoma and positively regulates skeletal muscle formation [20,21]. RSPO2 has also been reported to be highly expressed in many human organs, including the brain [22]. In the present study, the expression of the dopaminergic neuron marker (*TH*) and *MAP2* was detected by RT-PCR analysis of the neuro-organoids at day 61 (Figure 6A).

In the NE phase, bFGF and EGF were added to the DN medium for neuro-organoid culture. In general, bFGF and EGF promote the proliferation, survival, and self-renewal of neural stem cells and progenitor cells [23,24]. In particular, bFGF has been reported to stimulate the development of astroglial and oligodendroglial cells, facilitate neural cell survival, and promote neurite outgrowth [25]. As a result, the neuro-organoids were cultured until day 61 and the expression of MAP2, PAX6, S100, SOX2, and Nestin was confirmed by Immunofluorescence staining (Figure 6D). This indicates that multiple stages of neural cells are present together in a single neuro-organoid.

For long-term culture of neuro-organoids, Y-27632. was used for one day before NI step. According to previous reports, treatment with Rock inhibitor (Y-27632) on human ESCs reduces dissociation-induced apoptosis. In addition, it has been reported that apoptosis is prevented in the formation of suspended aggregates in SFEB culture [26]. In this study, the survival rate of suspended aggregates was also increased (Figure 7B). In addition, a medium in which low-glucose DMEM and F10 were mixed in 1:1 was used as the basal medium. It is mainly used as the basal medium for pESLCs. Using this, it was cultured for up to 102 days when culturing neuro-organoids. Furthermore, the protein content of the early neuronal marker NESTIN and the mature neuronal marker MAP2 was confirmed to have increased (Figure 7C). From this, it seems more suitable to use Y-drug and pESLC medium for long-term culture of porcine neuro-organoids.

Our study is a 3D neuro-organoid formation study using pESLC. Recently, Choi et al., used pEGCs to generate neural precursor cells [9]. Compared to our study, the initial SFEBq method is the same, but using a commercial medium (STEMdiff^TM^ Neural Induction Medium (STEMCELL Technologies, Vancouver, BC, Canada)), the composition is different from that of ours, and this product will be suitable for human cells. In addition, after the NI step, the plate was re-seeded and characterized under 2D conditions. However, we form 3D neuro-organoids by going through an aggregation process using ECM. After that, it went through the steps of NP and NE step. In addition, it was analyzed as it is in the 3D structure. Therefore, this is the first study of 3D neuro-organogenesis using a pESLC line. Processing of RSPO2 in the NP stage can cause differentiation toward the midbrain. The pESLC lines show similar characteristics to human ESCs. This technology will be useful for promoting research on the physiology of astrocytes and for producing CNS disease models such as Parkinson disease, Huntington disease, Alzheimer disease, and brain tumors.

## 4. Materials and Methods

### 4.1. Ethics Statement

The experimental protocol was approved by the Committee on Ethics of Animal Experiments of the Chungbuk National University (Permit Number: CBNUA-1460-20-02).

### 4.2. Oocytes Collection and In Vitro Maturation (IVM)

Oocyte collection and IVM were performed according to the methods described by Hwang et al. [27]. In brief, porcine ovaries were collected from the slaughterhouse (Dong-A Food, Cheongju, Korea), which were cultured at 37 °C in a 0.9% (wt/vol) NaCl solution and transferred to the laboratory. Using an 18-g needle and syringe, cumulus oocyte complexes (COCs) were aspirated with porcine follicular fluid (pFF) in 3–6-mm follicles. COCs were graded under the stereomicroscope and cultured in a 4-well dish (Nunc, Roskilde, Denmark) with 500 μL of maturation medium (0.6 mM cysteine, 0.91 mM sodium pyruvate, 10 ng/mL epidermal growth factor (EGF), 75 μg/mL kanamycin, 1 μg/mL insulin, and 10% (*v*/*v*) pFF in TCM 199; Gibco, Grand Island, NY, USA). For the first 22 h, 10 IU/mL equine chronic gonadotropin (eCG) and 10 IU/mL human chorionic gonadotropin (hCG) (Intervet, Boxmeer, Netherland) were added to the IVM medium. Thereafter, eCG/hCG was not added for the next 20 h of maturation. IVM was performed at 39 °C in a humidified incubation with 5% CO_2_ (Astec, Fukuoka, Japan). After IVM for 42 h, mature COCs were denuded by gentle pipetting with 0.1% hyaluronidase. The obtained matured oocytes were used for subsequent experiments.

### 4.3. SCNT and In Vitro Culture (IVC)

The TG donor cell line (Yucatan EGFP) used for SCNT was obtained from Kim’s Lab (Korea University, Seoul, Korea). This is a Yucatan miniature pig cell line that shows EGFP expression. SCNT was performed by selecting only mature oocytes in metaphase II (MII). The MII oocytes were washed using calcium-free HEPES-buffered Tyrode’s medium (0.05% w/v) containing 0.2% bovine serum albumin (TLH-BSA). Enucleation was performed using a 16-mm glass pipette (Humagen, Origio, Charlostesville, VA, USA) from a micro-manipulator in TLH containing 5 μg/mL cytochalasin B. After enucleation, trypsinized TG donor cells were transferred into the perivitelline space of enucleated oocytes, which were then fused by two pulses with a direct current of 180 V/mm for 60 μs in a 260 mM mannitol solution containing 0.1 mM CaCl_2_ and 0.05 mM MgCl_2_ using a cell fusion generator (LF201; Nepa Gene, Chiba, Japan). After electrical fusion, the SCNT embryos were incubated in 30 µL porcine zygote medium (PZM) droplets with 6DMAP (0.4 μg/mL demecolcine and 6-dimethyl aminopurine) for 4 h post-activation. Finally, the embryos were transferred to PZM droplets for IVC. On the second day after fusion, embryo CL was evaluated (1-cell, 2–3-cell, 4–5-cell, 6–8-cell stages, and fragmented embryos) and transferred to new PZM droplets. On the 4th day, the embryos were transferred to PZM droplets containing 10% fetal bovine serum (FBS). On the 7th day after fusion, BL formation was evaluated quantitatively (early, expanded, and hatched BL).

### 4.4. Preparation of ICR Mouse Feeder Cells

Mouse embryonic fibroblasts (MEFs) used as feeder cell layers were prepared from ICR mouse strains. The fetuses were sacrificed from ICR mice at 13.5 days of pregnancy. The fetal head, intestines, and legs were physically removed, and the remaining fetal tissues were physically crushed in the presence of dPBS (WelGENE, Inc., Daegu, Korea), followed by washing by centrifugation twice at 2000 rpm for 2 min. The MEF culture medium was composed of Dulbecco’s modified Eagle medium (DMEM) containing 10% FBS, 1% non-essential amino acids, 1% glutamine, 0.1 mM β-mercaptoethanol, and 1% antibiotics-antimycotics (all from Gibco). MEFs were cultured at 37 °C in a 5% CO_2_ humid incubator (Astec). Mitomycin C (10 µg/mL, Roche, Basel, Switzerland) was added to the MEF for 2–2.5 h to produce mitotically inactive ICR MEF feeder cells. These feeder cells were then plated at a density of 5 × 10^5^ cells/mL in a 4- or 6-well dish coated with 0.5% gelatin (WelGENE, Inc.) containing MEF culture medium. The ICR MEF feeder cells were typically plated 1–2 days before seeding of porcine BLs or ESLC.

### 4.5. Culture of pESLCs

On day 7 after SCNT, the BLs were seeded onto ICR MEFs at 5 × 10^5^ cells/mL. The pESLC culture medium was supplemented with 15% FBS and 4 ng/mL basic fibroblast growth factor (bFGF; BioBud, Seoul, Korea) in low-glucose DMEM (11885; Gibco). The pESLC medium was changed every day, and subculture was performed every week. For subculture, only pESLC colonies were detached from the ICR MEF feeder cells, mechanically cut into several pieces, and placed on top of the ICR MEF feeder cells.

### 4.6. Alkaline Phosphatase (ALP) Activity Detection

Porcine ESLCs were washed three times with dPBS and fixed in 4% paraformaldehyde for 10 min at 25 °C. Tris solution (0.1 M Tris, NaCl, pH 9.48) and NBT/BCIP chromogen solution (Roche) were added to the wells containing pESLCs and incubated for 20–60 min. The cells were washed three times with Tris solution and observed under a microscope.

### 4.7. pESLC Aggregation and Neuro-Organoid Differentiation

For aggregation, only pESLC colonies were detached from ICR MEF cells and then mechanically cut into several pieces. These pieces were placed in an Ultra-Low Attachment 24-Well Plate (Corning^®^ Costar^®^ Ultra-Low Attachment 24-Well Plate; Corning, Inc., Corning, NY, USA) and cultured for 3 days in pESLC medium without bFGF. Neural induction (NI) I medium was supplemented with 15% KnockOut Serum Replacement (KSR; Gibco) in DMEM/F12 (Gibco) and additional dSMADi, 10 μM of SB431541 (a transforming growth factor-β inhibitor), and 100 nM LDN193189 (a BMP4 inhibitor; Stemgent Inc., San Diego, CA, USA). The neuro-organoids were incubated for 9 days, and NI I medium was added after pESLC aggregation. Subsequently, the NI II medium was supplemented with 1× N2 (Gibco) and 1 µg/mL heparin (STEMCELL Technologies) in DMEM/F12 medium. The neuro-organoids were incubated for 8 days in NI II medium. Experiments were conducted using DN medium comprising DMEM/F12 and neurobasal medium (Gibco) at a 1:1 ratio along with N2, B27 (Gibco), and 10 µg/mL insulin. In the gel-coating step, 2% Geltrex, a basement membrane matrix, was mixed with DN medium for 2 days. In the neural patterning (NP) step, 0.5 µM retinoic acid (RA), 500 ng/mL sonic hedgehog (SHH; PeproTech, Rocky Hill, NJ, USA), 50 ng/mL FGF8, and 100 ng/mL RSPO2 (3266-RS-025; R&D System, Minneapolis, MN, USA) were added to the DN medium and the neuro-organoids were cultured for 7 days. In the neural expansion (NE) step, RA, 10 ng/mL EGF, and 4 ng/mL bFGF (BioBud, Seongnam-si, Gyeonggi-do, Korea) were added to the DN medium, followed by incubation.

### 4.8. pESLC Aggregation and Neuro-Organoid Differentiation (for Long-Term Culture)

The pESLC piece cut in the same manner as before was put in an Ultra-Low Attachment 24-Well Plate and cultured for 1 day in MEF-CM medium to which 10 uM 10 Y-27632. was added. MEF-CM medium was prepared by incubating ICR MEFs in pESLC culture medium for 1 day and then filtering only the medium with a 0.2 um syringe filter (Corning). After that, low-glucose DMEM/F10 was used as the basal media used in the Neural induction I, II step. Other reagents are the same as before (M&M 4.7).

### 4.9. Immunofluorescence Staining

The neuro-organoids were incubated in 4% paraformaldehyde for 20 min at room temperature or overnight at 4 °C for fixation. The neuro-organoids were then washed at least three times with dPBS and added to a 30% sucrose-PBS solution in a microcentrifuge tube for overnight incubation at 4 °C. The neuro-organoids were washed 2–3 times with dPBS in the tubes, and then, all of the dPBS was removed. Optimal Cutting Temperature (OCT) compound (Leica, Wetzlar, Germany) was added to the microcentrifuge tube containing the neuro-organoids, and the neuro-organoid was placed in the center of the cryomold together with OCT compound using a pipette. The samples were frozen on liquid nitrogen for 30 min or until the tissue-freezing medium became opaque. The neuro-organoid blocks were cryo-sectioned into 10–15-µm-thick sections with a Leica CM1950 cryostat at −25 °C. The neuro-organoid sections were attached to histobond slides (Marienfeld Glassware, Bad Mergentheim, Germany), and the slide-affixed neuro-organoid sections were stored at −20 °C until immunofluorescence staining.

For immunofluorescence staining, slide-affixed neuro-organoid sections were encircled with PAP Pen as a hydrophobic barrier (ImmEdge™; Vector Laboratories, Burlingame, CA, USA). The neuro-organoid section slides were placed in a humid chamber, and all subsequent procedures were conducted at room temperature in the humid environment. The neuro-organoid sections were washed twice with dPBS for 5 min each time and fixed with 4% paraformaldehyde for 30 min, followed by washing three times with dPBS for 5 min each time and permeabilized with 0.2% Triton X-100 for 30 min, and then washed three times again with dPBS for 5 min each; the image was enhanced using Image-iT™ FX Signal Enhancer (Invitrogen, Carlsbad, CA, USA) for 30 min. Finally, the neuro-organoid sections were washed three times with dPBS for 5 min each time and then co-incubated with blocking solution (10% goat serum in PBS) and with the primary antibody overnight at 4 °C. The antibodies used in this study are listed in Table 1. On the following day, the neuro-organoid sections were washed three times with dPBS for 5 min each time and incubated with the appropriate secondary antibodies at room temperature for 1 h. The neuro-organoid sections were washed three times with dPBS for 5 min each time and nuclei were stained with Hoechst 33342, followed by washing three times with dPBS for 5 min each and mounting with anti-fade mounting medium (Molecular Probes, Inc., Eugene, OR, USA). The stained neuro-organoid sections were examined by confocal microscopy (Leica Microsystems, Bannockburn, IL, USA).

### 4.10. Genomic DNA and Total RNA Extraction

Genomic DNA was isolated from the frozen aggregates and cells using the G-spin Total DNA Extraction Kit (iNtRON Biotechnology, Seoul, Korea) according to the manufacturer’s instructions. Total RNA was extracted using TRIzol (Invitrogen, Carlsbad, CA, USA) according to the manufacturer’s instructions. cDNA was synthesized using Moloney murine leukemia virus reverse transcriptase (Invitrogen) and random primers (Takara Bio, Shiga, Japan).

### 4.11. RT-PCR and Gel Electrophoresis

The genomic DNA (gDNA) or cDNA was amplified in a 20-μL PCR sample containing 1 U of i-StarTaq DNA polymerase (Intron Bio, Seongnam, Korea), 1× PCR buffer (30 mM Tris-HCl (pH 9.0), 30 mM salts containing of K^+^ and NH_4_^+^, 2 mM MgCl_2_), 2 mM dNTP mix, and 10 pM of each gene-specific primer. PCR conditions were pre-denaturation at 95 °C for 5 min; 30 cycles of denaturation at 95 °C for 30 s, annealing at 58 °C for 30 s, and extension at 72 °C for 30 s, followed by 72 °C for 5 min. PCR products were analyzed by gel electrophoresis (Mupid-exU; Takara Bio) at 100 V for 20 min on a 1.25% agarose gel with RedSafe (iNtRON Biotechnology). Gel images were obtained using a Bio-1000F gel imager (Microtek International, Hsinchu, Taiwan, Republic of China). All of the primer sequences are presented in Table 2. RN18s is used as control. RN18s is a housekeeping gene like GAPDH and has been reported to be very stable gene in pigs [28].

### 4.12. Capillary Western Blots Analyses

Capillary Western blots analyses were performed using the ProteinSimple Wes System (Protein Simple, New York, NY, USA). All procedures were performed according to the 12–230 kDa Wes Separation Module, 8 × 25 capillary cartridges (SM-W004-1, Protein Simple) kit protocol. Briefly, the total proteins from samples were extracted using RIPA lysis buffer (ProEX CETi lysis buffer, TransLab, Korea). The protein concentration was measured using the BCA (bicinchoninic acid) method. For this, 5 × Fluorescent Master Mix was added and heated at 95 °C for 5 min. After this denaturation step, sample, biotinylated ladder, blocking reagent, primary antibodies, HRP-conjugated secondary antibodies (Anti-Rabbit Detection Module, DM-001; Anti-Mouse Detection Module, DM-002; Protein Simple), and chemiluminescent substrate were dispensed into designated wells in an assay plate according to the kit protocol. After plate loading, the separation electrophoresis and immunodetection steps take place in the fully automated capillary system. Primary antibodies included anti-Oct4 (1:100 rabbit polyclonal, ab19857, Abcam, Cambridge, UK), anti-Nestin (1:100 mouse monoclonal, MAB5326, Millipore, Billerica, MA, USA), anti-MAP2 (1:100 rabbit polyclonal, 4542S, Cell Signaling Technology, Danvers, MA, USA), and anti-GAPDH (1:200, rabbit monoclonal, 2118S, Cell Signaling Technology). Data analysis is performed using the Compass Software Ver 5.0.1 (Build 0911; ProteinSimple).

## Figures and Tables

**Table 1:** Antibodies used for immunofluorescence staining.

Name	Host	Dilution	Cat #
OCT4	Rabbit	1:200	ab19857
Desmin	Mouse	1:200	MAB3430
Cytokeratin 17	Mouse	1:200	ab49749
Neurofilament	Mouse	1:200	MAB1615
Nestin	Mouse	1:200	MAB5326
PAX6	Rabbit	1:200	AB2237
S100B	Rabbit	1:200	ab868
Tuj1	Mouse	1:200	MAB1637
MAP2	Rabbit	1:200	4542S
Alexa Fluor 594	Anti-mouse	1:400	A11032
Alexa Fluor 594	Anti-rabbit	1:400	A21207

**Table 2:** Primer sequences used for gene analysis using PCR.

Gene	Primer Sequences (5′-3′)		Product Size (bp)	Accession Number
**RN18s**	F	CGCGGTTCTATTTTGTTGGT	219	NR_046261
	R	AGTCGGCATCGTTTATGGTC		
**pOct4**	F	AACGATCAAGCAGTGACTATTCG	153	AF074419
	R	GAGTACAGGGTGGTGAAGTGAGG		
**pNanog**	F	AATCTTCACCAATGCCTGAG	141	DQ447201
	R	GGCTGTCCTGAATAAGCAGA		
**pSox2**	F	CGGCGGCAGGATCGGC	113	EU519824
	R	GAGCTCCGCGAGGAAAA		
**Rex1**	F	TTTCTGAGTACGTGCCAGGC	201	TC206552
	R	GAACGGAGAGATGCTTTCTCAGAG		
**Nrob1**	F	CACGGCAGAGTGGCATCCT	319	NW_003612745
	R	CCAAGGTCTCCACCGTCT		
**Klf2**	F	CCACCTCTTCTAACTCAG	189	NW_003609596
	R	CTCTTGAACCGACTCAGCCT		
**bFGF**	F	GCGACCCTCACATCAAACT	214	AJ577089
	R	CAGTGCCACATACCAACT		
**bFGFR1**	F	ACTGCTGGAGTTAATACCACCG	125	AJ577088
	R	GCAGAGTGATGGGAGAGTCC		
**bFGFR2**	F	GGTGTTAACACCACGGACAA	139	AJ439886
	R	CTGGCAGAACTGTCAACCAT		
**AFP (Endo)**	F	CGCGTTTCTGGTTGCTTACAC	483	NM214317
	R	ACTTCTTGCTCTTGGCCTTGG		
**DESMIN (Meso)**	F	CCTCAACTTCCGAGAAACAAGC	108	NM1001535
	R	TCACTGACGACCTCCCCATC		
**CRABP2 (Ecto)**	F	CTGACCATGACGGCAGATGA	185	NM001164509
	R	CCCCAGAAGTGACCGAAGTG		
**EGFP**	F	AACGGCCACAAGTTCAGCGT	242	–
	R	TCACCTTGATGCCGTTCTTC		
**TH**	F	GCACGCCTCCTCGCCCAT	181	XM_021085452
	R	CTCCACCGTGAACCAGTA		
**MAP2**	F	GGGATTAGCAGTAACCCACG	224	XM_021075010
	R	AGGCCATCTGTCCAAAGTCA		

F: forward, R: reverse.

## Data Availability

The data presented in this study are available on request from the corresponding author.
